# Commercial Cu_2_Cr_2_O_5_ Decorated with Iron Carbide Nanoparticles as a Multifunctional Catalyst for Magnetically Induced Continuous‐Flow Hydrogenation of Aromatic Ketones

**DOI:** 10.1002/anie.202107916

**Published:** 2021-11-10

**Authors:** Hannah Kreissl, Jing Jin, Sheng‐Hsiang Lin, Dirk Schüette, Sven Störtte, Natalia Levin, Bruno Chaudret, Andreas J. Vorholt, Alexis Bordet, Walter Leitner

**Affiliations:** ^1^ Max Planck Institute for Chemical Energy Conversion 45470 Mülheim an der Ruhr Germany; ^2^ Laboratoire de Physique et Chimie des Nano-Objets. Université de Toulouse INSA UPS LPCNO CNRS-UMR5215 135 Avenue de Rangueil 31077 Toulouse France; ^3^ Institut für Technische und Makromolekulare Chemie RWTH Aachen University Worringerweg 2 52074 Aachen Germany

**Keywords:** continuous flow, heterogeneous catalysis, magnetic induction, multifunctional catalyst, selective hydrogenation

## Abstract

Copper chromite is decorated with iron carbide nanoparticles, producing a magnetically activatable multifunctional catalytic system. This system (ICNPs@Cu_2_Cr_2_O_5_) can reduce aromatic ketones to aromatic alcohols when exposed to magnetic induction. Under magnetic excitation, the ICNPs generate locally confined hot spots, selectively activating the Cu_2_Cr_2_O_5_ surface while the global temperature remains low (≈80 °C). The catalyst selectively hydrogenates a scope of benzylic and non‐benzylic ketones under mild conditions (3 bar H_2_, heptane), while ICNPs@Cu_2_Cr_2_O_5_ or Cu_2_Cr_2_O_5_ are inactive when the same global temperature is adjusted by conventional heating. A flow reactor is presented that allows the use of magnetic induction for continuous‐flow hydrogenation at elevated pressure. The excellent catalytic properties of ICNPs@Cu_2_Cr_2_O_5_ for the hydrogenation of biomass‐derived furfuralacetone are conserved for at least 17 h on stream, demonstrating for the first time the application of a magnetically heated catalyst to a continuously operated hydrogenation reaction in the liquid phase.

## Graphical abstract

A commercial heterogeneous catalyst is decorated with iron carbide nanoparticles to produce a multifunctional catalytic system with magnetic heating capabilities. Under magnetic induction, local hot spots are generated at the catalyst surface, unlocking continuous‐flow hydrogenation of aromatic ketones under mild conditions. This approach is versatile and potentially transferrable to a wide range of heterogeneous catalysts and transformations.

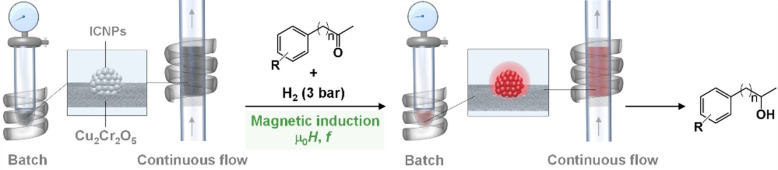

## Introduction

**Figure 1:**
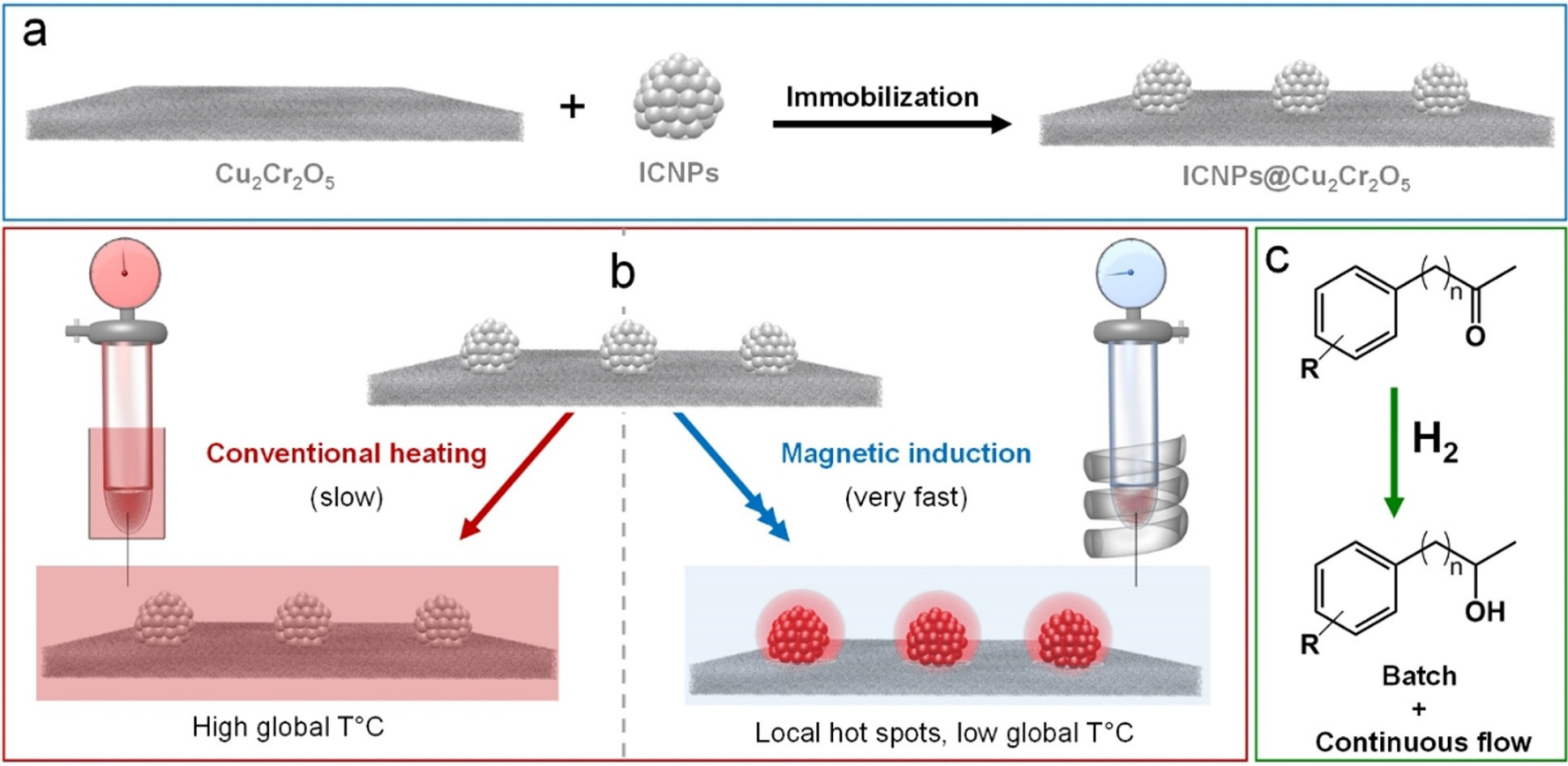
Illustration of the approach followed in this study. a) Preparation of the ICNPs@Cu_2_Cr_2_O_5_ catalyst, b) activation of the catalyst through conventional heating versus magnetic induction, c) catalytic application.

The generation of heat by magnetic materials exposed to high frequency alternating current magnetic fields is a well‐known phenomenon that has been used for decades in metallurgy, heating plates, and various biomedical applications.^[1]^ Attempts were also made to exploit the benefits associated with magnetic induction in heterogeneous catalysis.^[2]^ More recently, small magnetic nanoparticles heated by magnetic induction were used either as heating agents or directly as catalysts to effectively activate chemical transformations in the gas phase (e.g., Fischer–Tropsch,^[3]^ CO_2_ methanation,^[4]^ propane dehydrogenation,^[4e]^ methane steam reforming^[5]^) as well as in solution (e.g., hydrogenation,^[6]^ amidation,^[7]^ water splitting,^[8]^ hydrogenolysis^[9]^). Under magnetic induction, the heat required to unlock catalytic activity is generated directly by the magnetic nanocatalysts (Figure 1). This phenomenon is associated with potential benefits including energy efficiency, localized heating, rapid heating and cooling, and adaptivity to intermittent energy supply,[^4a^, ^10^] thus offering promising new perspectives for process intensification. These technical aspects may translate into favorable economics of a process such as downsizing of equipment, energy savings, or higher space/time yields.

The efficiency of the inductive heating—commonly defined by the Specific Absorption Rate, SAR of the receiving material—is intimately linked to the properties of the magnetic nanoparticles used (size, composition, shape, crystallinity, etc.).^[11]^ Recently, iron carbide nanoparticles (ICNPs) of tailor‐made size and composition^[12]^ were demonstrated to possess exceptionally high SAR to perform high temperature catalytic reactions through magnetic induction.^[4a]^ Using ICNPs as heat source to activate small Ru or Ni NPs, excellent catalytic properties have been observed for the hydrogenation of carbon dioxide to methane.^[4]^ In addition, ICNPs decorated with small Ru nanoparticles were found active for the magnetically induced hydrodeoxygenation of acetophenone derivatives under low hydrogen pressure (3 bar H_2_) in batch reactor, giving the corresponding aromatic alkane products in poor to high yields depending on the substituents.^[6a]^

While there is a rapidly growing interest in using magnetically heated nanoparticles in catalysis, it is fair to say that the potential of magnetic induction in catalysis has only been tapped on until now. The approach is still associated with severe challenges and limitations, including for example tedious catalyst synthesis procedures, fast deactivation/agglomeration during catalysis, and inapplicability under continuous flow conditions.[^4^, ^5^, ^6^] As a result, the benefits of magnetic induction are until now only accessible using complex and specifically designed materials. The development of versatile and stable catalytic systems beneficiating from magnetic induction heating is thus highly desirable for the generalization of this technique.

In this context, we report the preparation, characterization, and continuous‐flow application of a multifunctional catalytic system composed of ICNPs‐decorated copper chromite (ICNPs@Cu_2_Cr_2_O_5_, Figure 1).

The commercial heterogeneous catalyst Cu_2_Cr_2_O_5_ was decorated with magnetic ICNPs using a straightforward procedure. The ICNPs were synthesized following an organometallic approach and immobilized on the solid catalyst through a method involving impregnation and heat fixation under magnetic induction (Figure 1 a). Characterization of the resulting ICNPs@Cu_2_Cr_2_O_5_ material was achieved using a variety of techniques including electron microscopy, ICP, XRD, and magnetic measurements. The hydrogenation of benzylic and non‐benzylic ketones was selected as case study to investigate the catalytic properties of ICNPs@Cu_2_Cr_2_O_5_ activated by magnetic induction under batch and continuous flow conditions (Figure 1 b,c). This transformation is important not only for the conversion of biogenic feedstocks and platform chemicals but also for the production of fine chemicals and pharmaceuticals.^[13]^ A particular focus was placed on the comparative performances of ICNPs@Cu_2_Cr_2_O_5_ activated by conventional heating and magnetic induction, as well as on the catalyst stability and applicability under continuous flow conditions.

## Results and Discussion

Iron carbide nanoparticles (ICNPs, Fe_2.2_C@Fe_5_C_2_ core@shell structure) of average size 14 nm were prepared according to a procedure previously developed by some of us.^[4a]^ In brief, this involved first the organometallic synthesis of size‐controlled zero‐valent iron nanoparticles, followed by their controlled carbidization under CO/H_2_ (see Supplementary Information for details). In a second step, ICNPs were dispersed in THF and the colloidal solution obtained was added to Cu_2_Cr_2_O_5_ (targeted loading: 12 wt % ICNPs=> ca. 9 wt % Fe). The resulting mixture was sonicated under argon at r.t., leading to a black precipitate and a clear supernatant after 5 min. At the end of this impregnation step, the precipitate was dried under vacuum, giving a magnetic black powder. Finally, the resulting material was treated using magnetic induction (*μ_0_* H_max_ ≈45 mT, 350 kHz) for 1 h, thereby “anchoring” the ICNPs to the Cu_2_Cr_2_O_5_ surface. Without such treatment, ICNPs leaching and poor catalytic performance were observed, as discussed later.

**Figure 2:**
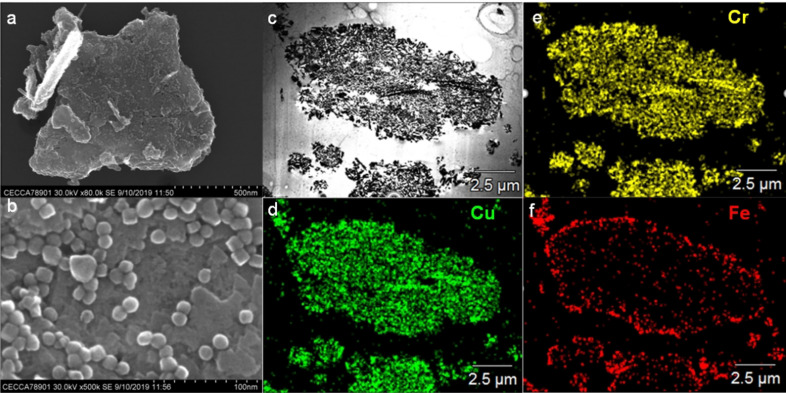
Characterization of ICNPs@Cu_2_Cr_2_O_5_ by electron microscopy. a,b) SEM images at different resolutions; c) SEM image on ICNPs@Cu_2_Cr_2_O_5_ prepared by microtomy; d–f) SEM‐EDX elemental mapping of d) Cu, e) Cr and f) Fe.

Characterization of the ICNPs by transmission electron microscopy (TEM), powder X‐ray diffraction (XRD) and vibrating sample magnetometer (VSM) provided information about the NPs size (13.7 +/− 1.0 nm), crystallinity (pseudo‐hexagonal Fe_2.2_C) and magnetic properties (*M_S_* =140 emu g^−1^), respectively (Figure S1–3). In addition, measurements of specific absorption rates (SARs) by calorimetry evidenced the very high heating power of ICNPs under magnetic induction (ca. 2500 W g^−1^ at *μ_0_* H_max_=66 mT, *f*=100 kHz, Figure S4). These characterization data of ICNPs are consistent with the results described in a prior publication.^[4a]^ Analysis of the ICNPs@Cu_2_Cr_2_O_5_ by scanning electron microscopy with energy dispersive X‐ray spectroscopy (SEM‐EDX) evidenced evenly distributed ICNPs over the Cu_2_Cr_2_O_5_ surface (Figure 2).

The Fe loading of ICNPs@Cu_2_Cr_2_O_5_ was determined to be 8.9 wt % by inductively coupled plasma optical emission spectroscopy (ICP‐OES, Table S1), well in agreement with the theoretical value. Characterization of ICNPs@Cu_2_Cr_2_O_5_ by powder XRD gave characteristic patterns for Cu_2_Cr_2_O_5_ for both its unreduced and reduced state (Figure S5), in accordance with literature.^[14]^ Diffraction peaks associated to the ICNPs could not be clearly identified in ICNPs@Cu_2_Cr_2_O_5_, presumably due to peak overlapping of the characteristic regions with those of Cu_2_Cr_2_O_5_ (Figure S6), as well as to a relatively low loading of ICNPs.

**Scheme 1:**
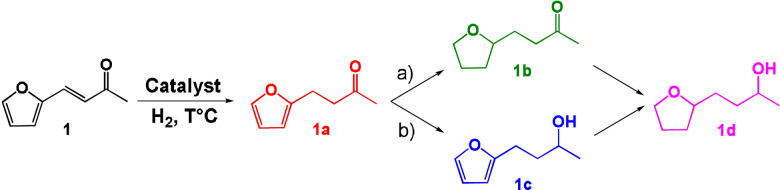
Hydrogenation network of furfuralacetone (**1**).

The catalytic properties of ICNPs@Cu_2_Cr_2_O_5_ were first investigated for the hydrogenation of furfuralacetone (FFA) as model substrate. This choice was motivated by the multiple hydrogenation steps of FFA (Scheme 1), as well as by its potential as a source for biomass utilization and future industry applications.^[15]^

The first step of the reaction network consists in the hydrogenation of the C=C bond to give 4‐(2‐furanyl)‐2‐butanone (**1 a**). Then, either the furan ring is hydrogenated first, giving the saturated ketone 4‐(tetrahydro‐2‐furanyl)‐2‐butanone (**1 b**) through route a), or the ketone is hydrogenated first, giving the unsaturated alcohol α‐methyl‐2‐furanpropanol (**1 c**) through route b). Finally, **1 b** and **1 c** are hydrogenated to form the completely saturated tetrahydro‐α‐methyl‐2‐furanpropanol (**1 d**). Cu_2_Cr_2_O_5_ is known to selectively reduce ketones without hydrogenating aromatic rings,[^13b^, ^16^] and is thus expected to follow pathway b) and stop at product **1 c** without further hydrogenation of the furan ring.

The catalytic performance of ICNPs@Cu_2_Cr_2_O_5_ was first investigated in batch conditions using Fischer‐Porter bottles as reactors (Table 1). In typical experiments, furfuralacetone (**1**, 13.7 mg; 0.1 mmol) was dissolved in heptane (0.5 mL) and reacted in the presence of the catalyst (30.0 mg) under H_2_ (3 bar) and magnetic induction (350 kHz, *μ_0_* H_max_=42–80 mT) for 2 h. For comparison, the results obtained from reactions performed with classical heating under the same global temperature are also given (Table 1 Entry 5–8). No stirring was implemented and mixing was insured only through convection. The ICNPs@Cu_2_Cr_2_O_5_ catalyst was well dispersed in the reaction media, forming a fairly homogeneous suspension. For the different field amplitudes applied, the global temperature of the reaction solution resulting from the heat dissipated by the ICNPs was measured using an infrared camera. This, in addition to a local temperature estimation method, allowed for the setting up of a magnetic field amplitude to global temperatures correlation (Table S2).

**Table 1:** Hydrogenation of furfuralacetone (**1**) using ICNPs@Cu_2_Cr_2_O_5_ activated by magnetic induction or classical heating.

	Entry	*μ_0_* H_max_ [mT]	Global Temp. [°C]	Conv. [%]	Product Yield [%]	
					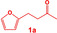	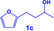
Magnetic induction	1	42	40	41	41	0
	2	64	80	>99	9	91
	3	71	100	>99	0	>99
	4	80	120	>99	0	>99
Classical heating	5	–	40	0	0	0
	6	–	80	0	0	0
	7	–	100	33	33	0
	8	–	120	>99	2	98

Reactions performed in Fischer‐Porter bottle, with 30 mg ICNPs@Cu_2_Cr_2_O_5_ + 0.1 mmol **1** in 0.5 mL heptane under 3 bar H_2_ for 2 h without stirring. Product yield determined by GC‐FID using tetradecane as internal standard. H_2_ conversion at >99 % yield of **1 c**=ca. 4 %.

At low field strength, the conversion was incomplete, and only C=C hydrogenation leading to product **1 a** was observed (Entry 1). Increasing the amplitude of the magnetic field to 64 mT and above lead to the full conversion of the substrate and to the production of **1 c** in 91 % to >99 % yield. Thus, the activation by magnetic induction of ICNPs was found to be efficient to activate the Cu_2_Cr_2_O_5_ hydrogenation catalyst in solution. Interestingly, similar reactions conducted with classical heating at the same global temperature of 40 and 80 °C gave no conversion at all (Table 1 Entry 5 and 6). Raising the temperature to 100 °C led only to the hydrogenation of the C=C, giving **1 a** in poor yield (33 %). Full conversion to product **1 c** could only be observed when raising the temperature to 120 °C.

This confirms that under magnetic induction local hot spots allow the reaction to proceed at temperatures significantly higher than the observable global temperature of the reactor. It should be noted that the decoration with ICNPs did not have a significant impact on the intrinsic catalytic performance of Cu_2_Cr_2_O_5_. Comparable catalytic results were reached if the reactions with classical heating were performed with pure Cu_2_Cr_2_O_5_, demonstrating that Cu_2_Cr_2_O_5_ itself is the active catalyst. Along the same line, using only magnetically activated ICNPs without Cu_2_Cr_2_O_5_ did not lead to any furfuralacetone conversion (Table S3). Attempts to use a lower loading of ICNPs on Cu_2_Cr_2_O_5_ (5 wt %) under optimized conditions (64 mT, 2 h) led to very low conversions, presumably due to an insufficient heating of the catalyst (Table S4). On the other hand, increasing the loading of ICNPs to 20 wt % resulted only in a slight increase in the yield of **1 c** (95 % instead of 91 %). A loading of 12 wt % was thus conserved for the rest of the study.

**Figure 3:**
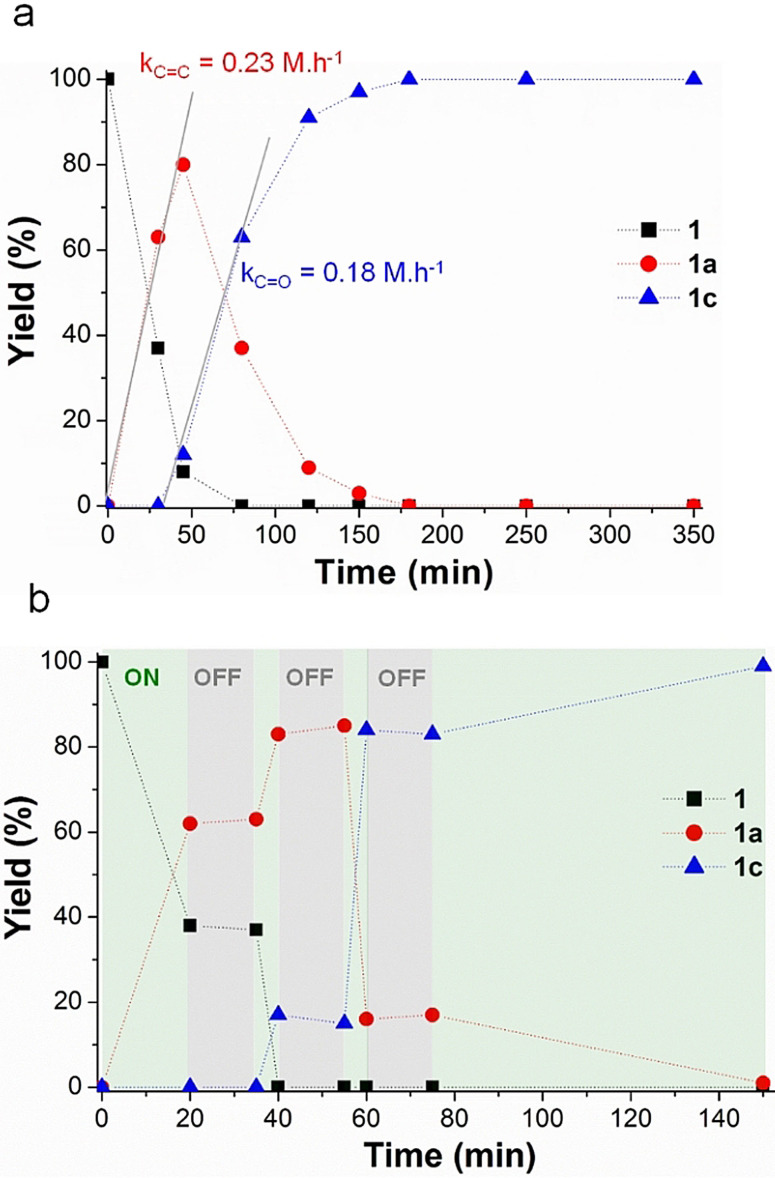
Kinetic study of the hydrogenation of **1** using ICNPs@Cu_2_Cr_2_O_5_ and magnetic induction at a field amplitude of 64 mT (global temperature ≈80 °C). a) time profile; b) time profile recorded while regularly switching ON and OFF the power supply of the magnetic induction generator (green zones=power ON, grey zones=power OFF). Reactions performed in a Fischer‐Porter bottle, with 30 mg ICNPs@Cu_2_Cr_2_O_5_ + 0.1 mmol substrate in 0.5 mL heptane under 3 bar H_2_ without stirring. Yields determined by GC‐FID using tetradecane as internal standard.

To get further insight into the kinetics of the reaction, time profiles were recorded for the hydrogenation of furfuralacetone using ICNPs@Cu_2_Cr_2_O_5_ under magnetic heating at 64 mT (global temperature ≈80 °C) (Figure 3 a). The results show that the reaction proceeds first through the hydrogenation of the C=C to give **1 a**, followed by C=O hydrogenation to give a quantitative yield of **1 c** after 3 h without further hydrogenation of the aromatic ring, in a typical kinetic profile for sequential steps. The initial rates for C=C (k_C=C_=0.23 mol L h^−1^) and C=O (k_C=O_=0.18 mol L h^−1^) hydrogenation are very close, outlining the excellent ketone reduction activity of the ICNPs@Cu_2_Cr_2_O_5_ catalyst. Interestingly, stopping and re‐starting the alternating current magnetic field regularly during the recording of a time profile lead to the perfectly concomitant stop or re‐start of the catalyst activity (Figure 3 b). This outlines the extremely quick heating and cooling of the active sites provided by magnetic induction activation, and the catalytic system's very good suitability and adaptability to intermittent power supply. In agreement with the results discussed earlier, no significant conversion of **1** was observed when running the reaction with conventional heating at the same global temperature of 80 °C. A similar profile with comparable C=C and C=O hydrogenation activities could be observed only when raising the global temperature to 120 °C (Figure S7a), which is ca. 40 °C higher than the global temperature measured during the experiments under magnetic induction. In sharp contrast with the use of magnetic induction, conventional heating did not allow quickly stopping and re‐starting the reaction by switching ON and OFF the power supply due to much longer heating and cooling times (Figure S7b).

The versatility of the ICNPs@Cu_2_Cr_2_O_5_ catalyst under magnetic induction was explored for a scope of aromatic ketones (Table 2). Under optimized conditions, both non‐benzylic and benzylic ketone derivatives were readily hydrogenated to the corresponding aromatic alcohols in excellent yield and selectivity. The possibility to easily isolate the products was demonstrated for substrates **1** and **2**, giving the corresponding isolated aromatic alcohols in 95 % and 98 % yield, respectively. The presence of electron donating or withdrawing substituents in acetophenone derivatives did not influence significantly the activity and selectivity observed under these conditions (Substrates **5**–**8**). Interestingly, 2‐acetonaphtone (**9**) and benzophenone (**10**) were partially hydrodeoxygenated under standard conditions, suggesting that the ICNPs@Cu_2_Cr_2_O_5_ catalyst is active for C−O bond cleavage.

**Table 2:** Magnetically induced hydrogenation of benzylic and non‐benzylic ketones using ICNPs@Cu_2_Cr_2_O_5_. 
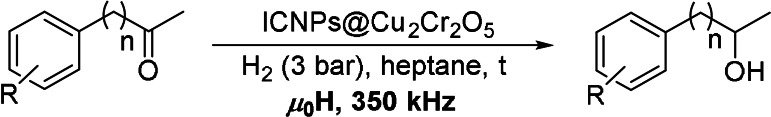

Entry	Substrate	*μ_0_* H_max_ [mT]	*t* [h]	Conversion [%]	Product yield [%]^[a]^
1	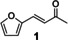	64	4	>99	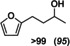
2	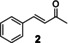	64	4	>99	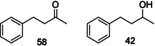
		64	8	>99	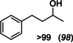
3	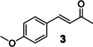	64	8	>99	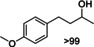
4	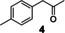	80	18	>99	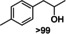
5	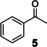	64	4	>99	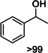
6	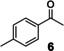	64	4	>99	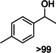
7	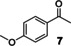	64	4	>99	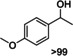
8	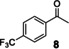	64	4	>99	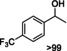
9	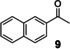	64	4	>99	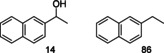
10	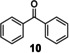	64	4	>99	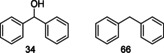

Reactions performed in Fischer‐Porter bottle, with 30 mg ICNPs@Cu_2_Cr_2_O_5_ + 0.1 mmol substrate in 0.5 mL heptane under 3 bar H_2_ for *x* h without stirring. [a] Determined by GC‐FID using tetradecane as internal standard. H_2_ conversion at >99 % yield of desired product=ca. 4 %. (*Isolated yield*)

These results are in contrast to what was observed using Ru@ICNPs catalysts, where benzylic ketones with electron donating substituents were hydrodeoxygenated to aromatic alkanes while substrates with electron withdrawing groups resulted in lower conversions.^[6a]^ Interestingly, aromatic alcohols were not accessible with this catalytic system.

In comparison, no ketone reduction activity was observed when performing the hydrogenation of non‐benzylic and benzylic ketones with classical heating at the same global temperature of 80 °C (Table S4). In the case of non‐benzylic ketones, comparable product yields could be obtained only when raising the temperature to 150 °C. Applying high temperatures (120 °C or 150 °C) with benzylic ketones led to the full hydrodeoxygenation of the substrates (Table S5). Interestingly, despite fairly comparable ketone hydrogenation activities of ICNPs@Cu_2_Cr_2_O_5_ at 64 mT and at 120–150 °C, selective hydrogenation of benzylic ketones is favored under magnetic induction while conventional heating leads to hydrodeoxygenation. This may suggest an influence of the heating mode on the reactivity of the ICNPs@Cu_2_Cr_2_O_5_ catalyst.

**Figure 4:**
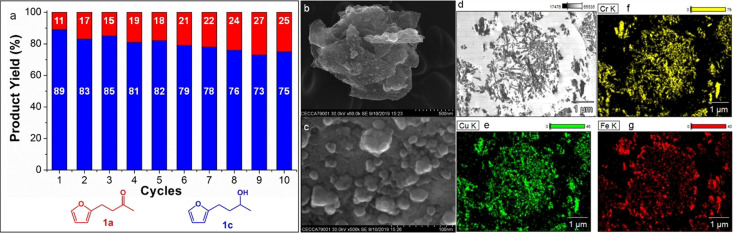
Study of the stability of ICNPs@Cu_2_Cr_2_O_5_ through recycling experiments. a) Catalytic results; b–g) Characterization of the catalyst by electron microscopy after the 10^th^ cycle: b–c) SEM images at different resolutions; d) SEM image on ICNPs@Cu_2_Cr_2_O_5_ prepared by microtomy; e–g) SEM‐EDX elemental mapping of e) Cu, f) Cr and g) Fe. Reactions performed in Fischer‐Porter bottle, with 30 mg ICNPs@Cu_2_Cr_2_O_5_ + 0.1 mmol substrate in 0.5 mL heptane under 3 bar H_2_ for 2 h at 64 mT without stirring.

The possibility to recycle the ICNPs@Cu_2_Cr_2_O_5_ catalyst was studied using furfuralacetone (**1**) as substrate. For this, the reaction conditions were selected to have a mixture of products **2** and **4**, allowing to probe any change in performance. The catalyst could be reused at least 10 times with fairly constant activity and selectivity (Figure 4), highlighting the excellent stability of both its catalytically active and magnetically heating components (Cu_2_Cr_2_O_5_ and ICNPs, respectively). The slight decrease of selectivity through the cycles is presumably due to the loss of small quantities of catalyst during the washing step applied between each cycle. SEM and SEM‐EDX characterization after 10 cycles showed no significant change of the catalyst surface. ICNPs are still evenly distributed over the Cu_2_Cr_2_O_5_ surface (Figure 4 b–g) and only minor deformations of the ICNPs can be noticed (Figure 4 c). Elemental analysis by ICP‐OES did not evidence any leaching of the Cu or Cr during the reaction (Table S1). Small amounts of ICNPs were removed from the catalyst after the first cycle (≈10 %). After this initial loss, no further ICNPs leaching was observed even after 9 additional consecutive experiments (Table S1). Interestingly, a ICNPs@Cu_2_Cr_2_O_5_ catalyst prepared without treatment under magnetic induction to anchor the ICNPs was found poorly recyclable (Table S6), presumably due to ICNPs detaching from the Cu_2_Cr_2_O_5_ surface during the reaction and workup. The performances and stability of the ICNPs@Cu_2_Cr_2_O_5_ catalyst were further investigated under continuous flow conditions using furfuralacetone (**1**) as substrate.

**Figure 5:**
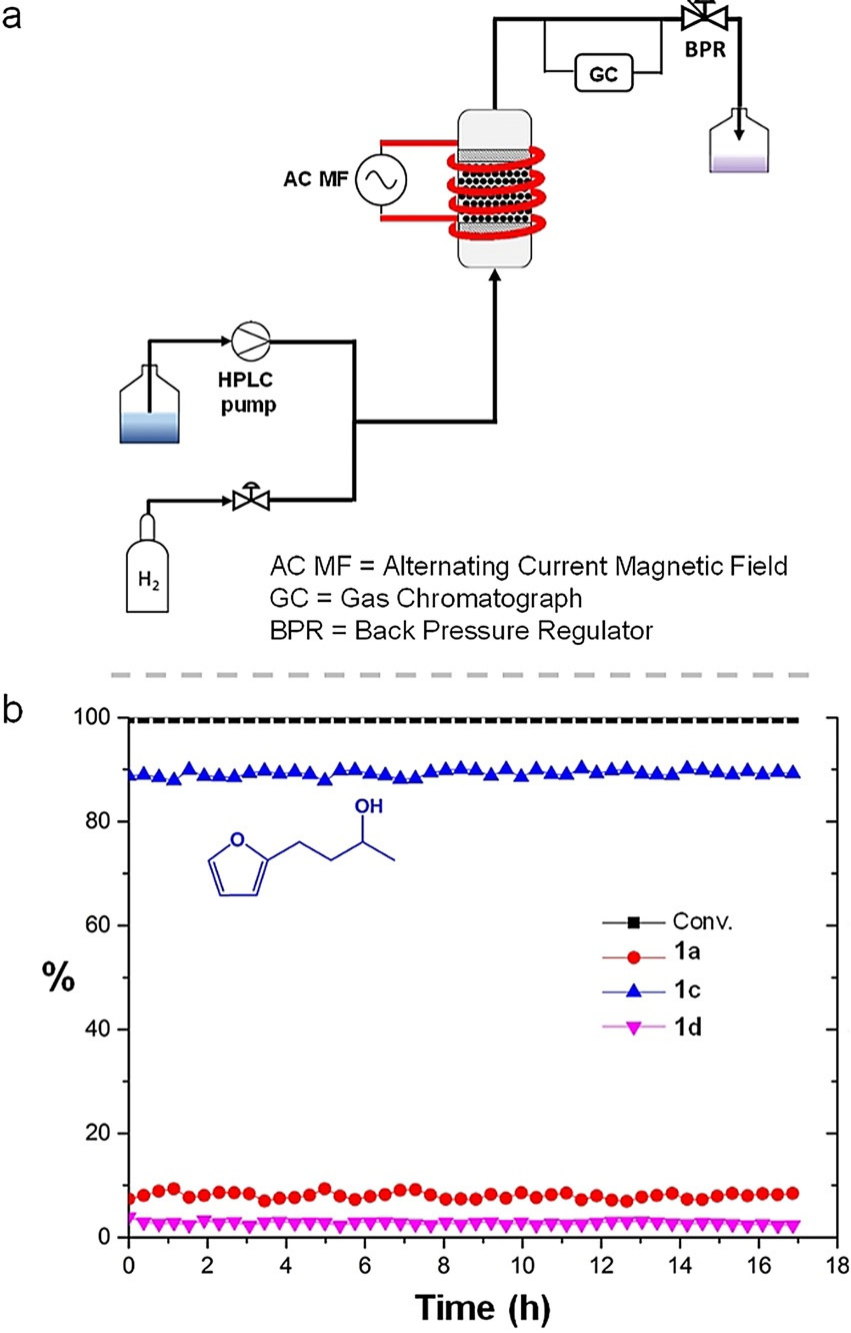
Continuously operated hydrogenation of furfuralacetone using ICNPs@Cu_2_Cr_2_O_5_ and magnetic induction. a) Simplified schematic representation of the continuous flow miniplant; b) catalytic results. Reaction conditions: the experiment was carried out in a continuous flow miniplant, with 500 mg of the ICNPs@Cu_2_Cr_2_O_5_ catalyst. Substrate **1** (0.05 mol L^−1^ in heptane) was pumped with a flow rate of 0.1 g min^−1^ and mixed into a flowing stream of H_2_ (0.001 g min^−1^). The magnetic field amplitude was set to 42 mT (350 kHz) and the system pressure was kept at 5 bar. WHS*V*=0.1 h^−1^ (0.1 g min^−1^). Liquid residence time=8.2 min. Product yield determined by a calibrated online GC‐FID. H_2_ conversion=3 %.

The development of a continuous flow set‐up able to accommodate our catalytic system (solid catalyst) and its operating conditions (magnetic induction=> metal‐free reactor; possibility to work up to 150 °C, liquid substrate solution; hydrogen pressure) is particularly challenging and has not been achieved until now. Figure 5 a shows a simplified schematic representation of our in‐house built continuous flow miniplant that allows the use of magnetic induction heating. In brief, the substrate solution (**1**, 0.05 mol L^−1^ in heptane, flow rate 0.1 g min^−1^, 0.15 mL min^−1^) is mixed into a flowing stream of H_2_ (0.001 g min^−1^, 2.47 mL min^−1^) resulting in a well‐mixed gas/liquid reactant phase entering the reaction zone. The resulting mixture flows through a high pressure HPCL glass column used as a tube reactor which contains a catalyst bed of 500 mg ICNPs@Cu_2_Cr_2_O_5_ (catalyst particle sizes ca. 1 μm, pressure drop <1 bar) and is subjected to the AC magnetic field. The system pressure is set to 5 bar and controlled by a back pressure regulator. At the output of the reactor, an online GC‐FID is installed to analyze the products in real‐time. A detailed description is provided in the Supplementary Information, Figure S8–9. After a rapid screening of the magnetic field amplitude (Table S7), the reaction conditions were fixed to *μ_0_* H_max_=42 mT, with a substrate flow of 0.1 g min^−1^ (0.15 mL min^−1^, liquid residence time=8.2 min, shorter than in batch) a gas flow of 0.001 g min^−1^ (2.47 mL min^−1^) and a pressure of 5 bar, WHS*V*=0.1 h^−1^. This gave complete conversion of the substrate and product **1c** in 88 % yield, corresponding to a H_2_ conversion of 3 %. A large excess of H_2_ (similar to the batch experiments) was used to avoid a significant gradient of the partial H_2_ pressure along the reactor bed. Under these conditions, a temperature of ca. 110 °C was measured by an infrared camera at the surface of the reactor. Raising the magnetic field amplitude above 42 mT lead to an increase of the global temperature (ca. 125 °C) but to a slight decrease of the catalytic activity (75 % of **1c**), presumably due to the local boiling of the solvent limiting the access of the substrate to the active sites. For comparison purposes, an analogous continuous flow experiment was performed using conventional heating at 120 °C instead of magnetic induction, by replacing the coil by a trace heater. Strikingly, no conversion of **1** was observed under these conditions, highlighting the advantage of using magnetic induction to perform a reaction locally at a temperature higher than the observable temperature measured at the surface of the reactor. The stability of the ICNPs@Cu_2_Cr_2_O_5_ catalyst under continuous flow conditions was studied for 17 h on stream under standard conditions (Figure 5 b). Excellent and constant activity and selectivity toward the formation of **1c** (ca. 90 % yield) were maintained at all time during the 17 h continuous flow reaction, without any sign of deactivation. This confirms and further demonstrates the remarkable and unprecedented stability and practicability of the ICNPs@Cu_2_Cr_2_O_5_ catalyst that was already glimpsed through the batch recycling experiments.

## Conclusion

In conclusion, we report the decoration of commercial Cu_2_Cr_2_O_5_ with magnetic iron carbide nanoparticles (ICNPs) of high heating power using a straightforward synthetic procedure. The resulting ICNPs@Cu_2_Cr_2_O_5_ multifunctional catalyst was applied to the hydrogenation of aromatic ketones to aromatic alcohols activated by magnetic induction. The reaction proceeded smoothly under mild conditions (3–5 bar H_2_, apparent reactor temperature ≈80 °C) without any additional heating thanks to the creation of local hot spots by the magnetically activated ICNPs on the Cu_2_Cr_2_O_5_ surface. Notably, the catalyst (decorated or not) is completely inactive under identical conditions applying conventional thermal heating. A scope of benzylic and non‐benzylic ketones was readily hydrogenated to the corresponding aromatic alcohols in excellent yields and selectivities. Recycling and long‐term experiments conducted under batch (10 cycles) and continuous flow conditions (17 h on stream) demonstrated effective reusability and high long‐term stability of the ICNPs@Cu_2_Cr_2_O_5_ catalyst. A continuous‐flow reactor for liquid‐phase reactions with pressurized gases was designed and built to tackle the challenges associated with the use of an alternating current magnetic field. To the best of our knowledge, this is the first report of a continuously operated liquid‐phase hydrogenation reaction catalyzed by a magnetically activated heterogeneous catalyst.

Exemplified here with the ICNPs@Cu_2_Cr_2_O_5_ catalyst for the hydrogenation of aromatic ketones, the presented strategy to design, synthesize, and apply multifunctional catalytic systems with magnetic heating capabilities should be transferrable readily to a wide range of heterogeneous catalysts and transformations. The study demonstrates the possibility to modify even standard heterogeneous catalysts with novel physical properties (e.g., extremely fast heating and cooling, local heating, energy efficiency, suitability to intermittent energy supply, etc.) in a simple and versatile manner. The presented methodology will enable a much broader exploration of the potential of magnetic induction in catalysis and may thus pave the way toward practical application of this innovative tool.

## Conflict of interest

The authors declare no conflict of interest.

## Supporting information

As a service to our authors and readers, this journal provides supporting information supplied by the authors. Such materials are peer reviewed and may be re‐organized for online delivery, but are not copy‐edited or typeset. Technical support issues arising from supporting information (other than missing files) should be addressed to the authors.

Supporting Information
