# Supplementary material for: Commercial Cu2Cr2O5 Decorated with Iron Carbide Nanoparticles as a Multifunctional Catalyst for Magnetically Induced Continuous‐Flow Hydrogenation of Aromatic Ketones
Source: Angew Chem Int Ed Engl. 2021 Nov 10;60(51):26639–46. doi: 10.1002/anie.202107916 (PMC9298693; doi:10.1002/anie.202107916)
Supplement: Supplementary file 1 — Supporting Information [file ANIE-60-26639-s001.pdf]

Angewandte Chemie
Eine Zeitschrift der Gesellschaft Deutscher Chemiker

Supporting Information

# Commercial $Cu_2Cr_2O_5$ Decorated with Iron Carbide Nanoparticles as a Multifunctional Catalyst for Magnetically Induced Continuous-Flow Hydrogenation of Aromatic Ketones

*Hannah Kreissl[+], Jing Jin[+], Sheng-Hsiang Lin[+], Dirk Schüette, Sven Störtte, Natalia Levin, Bruno Chaudret, Andreas J. Vorholt, Alexis Bordet,* and Walter Leitner*

anie_202107916_sm_miscellaneous_information.pdf

# Supporting Information

**General** ........................................................................................2
**Methods** ........................................................................................2
**Experimental** ........................................................................................2
Characterization ........................................................................................2
Synthesis ........................................................................................4
Iron carbide nanoparticles (ICNPs) ........................................................................................4
ICNPs@$Cu_2Cr_2O_5$ (12 wt% ICNPs loading) ........................................................................................4
Catalytic study ........................................................................................5
Conventional heating ........................................................................................5
Magnetic induction heating ........................................................................................5
Continuous flow miniplant ........................................................................................6
Continuous flow experiments ........................................................................................6
**Supplementary Tables and Figures** ........................................................................................7
**Isolated Yields** ........................................................................................15
Hydrogenation of furfuralacetone (**1**) ........................................................................................15
Hydrogenation of benzilideneacetone (**2**) ........................................................................................16

# General

Experiments involving magnetic induction and high-pressure of $H_2$ must be carried out only with appropriate equipment and under rigorous safety precautions.

# Methods

All synthetic procedures were carried out under argon using Schlenk techniques or the glovebox. Mesitylene (99%), toluene (99%), and tetrahydrofuran (99%) were purchased from VWR, heptane (99%) from Carl Roth and all solvents were degassed under argon and dried over molecular sieves (4 Å) before use. The commercial products, hexadecylamine (HDA), palmitic acid (PA) and copper chromite ($Cu_2Cr_2O_5$) were purchased from Sigma-Aldrich, the bis(amido)iron(II) dimer $\{Fe[N(SiMe_3)_2]_2\}_2$ from Nanomeps, acetophenone, 4-methylacetophenone, 4-methoxyacetophenone and 4-(trifluoromethyl)acetophenone from Alfa Aesar, 4-methylphenylacetone and benzylideneacetone from Sigma Aldrich. These compounds were stored in the glovebox and used without additional purification. Furfuralacetone was synthesized in-house by aldol condensation of furfural (Sigma Aldrich) and acetone with purification by sublimation, according to the previously published procedure.[1]

# Experimental

## Characterization

The size and the morphology of ICNPs (iron carbide nanoparticles) and of the preformed Fe(0) nanoparticles were studied by TEM (transmission electronic microscopy). TEM grids were prepared by drop casting a colloidal solution containing as prepared NPs on a copper grid covered with amorphous carbon. Conventional bright-field images were performed using JEOL microscopes (Model 1400 and 2010) working at 120 kV and 200 kV respectively. Fe(0) nanoparticles were shown to be of spherical or cubic shape with an average diameter of 12 nm (Figure S1a). Carbidization leads to an increase in particle size to about 14 nm for the ICNPs due to the incorporation of carbon (Figure S1b), which is in good agreement with the previously published results.[2] XRD (X-ray diffraction) measurements were performed on a PANalytical Empyrean diffractometer using Co-Kα radiation ($\lambda$=0.1789 nm) at 45 kV and 40 mA. The XRD patterns obtained for Fe(0) and ICNPs compare well to those reported[2] and confirm the presence of an iron carbide phase after carbidization (Figure S2). Magnetic measurements were performed on a VSM (Vibrating Sample Magnetometer, Quantum Device PPMS Evercool II). Plotting sample magnetization against applied field strength gives a saturation magnetization of ≈150 emu/g (Figure S3), comparable to the previously reported values.[2] SAR (Specific absorption rate) measurements to further determine the heating power of the ICNPs were conducted using the previously published calorimeter set-up. The SAR

values obtained in correlation with the applied field strength are in accordance with those reported previously (Figure S4).[2] For all XRD, VSM and SAR measurements air-tight sample holders were used and prepared in the glovebox. The iron content of ICNPs was determined by TGA (thermogravimetric analysis). TGA measurements were performed on a Mettler Toledo thermogravimetric analyzer using oxidation-reduction with a temperature ramp to 700°C to remove all carbon content from the sample. The samples were shown to contain ≈ 75 wt% iron, which is in good agreement with the previously published values.[2]

The size and the morphology of ICNPs@$Cu_2Cr_2O_5$ were studied by SEM (scanning electron microscopy) instead of TEM due to its better surface sensitivity and to avoid poor image contrast between the heavy elements (Fe,Cu,Cr). SEM and SEM-EDX measurements were performed by the microscopy department of the Max-Planck-Institut für Kohlenforschung.

ICP-OES (inductively coupled plasma optical emission spectroscopy) was performed by Kolbe Laboratories (Fraunhofer Institute UMSICHT Oberhausen) for elemental analysis of ICNPs@$Cu_2Cr_2O_5$ before and after catalysis. The experimental results were in line with the expected stoichiometry and further confirmed ICNP leaching of 11% over 10 consecutive runs (Table S1).

Expected element wt% for ICNPs@$Cu_2Cr_2O_5$ from ICNP loading and copper chromite stoichiometry: 12 wt% ICNPs (of which 75% = 9 wt% Fe as determined by TGA), 36 wt% Cu, 29wt% Cr, 23 wt% O. Comparison before and after catalysis: Fe wt% ratio after/before = 7.99/9.23 = 87%, Cu wt% ratio after/before = 36.27/37.09 = 98%, Cr wt% ratio before/after = 29.94/30.44 = 98%. 98% of initial composition for Cu/Cr most likely due to 2% coking/carbon depositions on catalyst surface, remaining deficit of 11% for Fe most likely due to Fe leaching from catalyst surface over consecutive reaction runs.

Powder XRD measurements of the catalysts were performed by the group of Claudia Weidenthaler at the Max-Planck-Institut für Kohlenforschung. To compare the structural changes occurring for pure $Cu_2Cr_2O_5$ during reduction to those reported in literature, an in situ reduction was performed of the commercial $Cu_2Cr_2O_5$ used (Figure S5). The observations were in accordance with those in literature.[3,4] Further, XRD spectra of ICNPs@$Cu_2Cr_2O_5$ were recorded in comparison to pure $Cu_2Cr_2O_5$, but unfortunately there was no obvious difference between the spectra (Figure S6), most likely due to the overlapping peak regions of Fe/$Fe_{2.2}C$ with $Cu_2Cr_2O_5$ and the relatively low loading of ICNPs.

Product analysis was done by GC-FID (gas chromatography coupled with flame ionization detection) on a Shimadzu GC 2030 equipped with a CP-WAX-52CB column and further by GC-MS (gas chromatography coupled with mass spectrometry) on a Shimadzu QP 2020 instrument. Samples obtained from the reaction mixture as described above were injected into

the GC-FID and were identified according to the respective product retention times. Product quantification was done by referencing the product peak area to the peak area of the added tetradecane standard, following internal GC calibration with the isolated products. For identification of unknown products and trace compounds GC-MS was used with its internal compound library for product identification.

## Synthesis

### *Iron carbide nanoparticles (ICNPs)*

ICNPs were synthesized according to the previously published procedure.[2] They were obtained in two steps through the carbidization of preformed Fe(0) nanoparticles:

Synthesis of Fe(0) nanoparticles

In the glove box, 0.65 mmol of PA (333.2 mg) and 0.5 mmol of HDA (241.5 mg) were each dissolved in 10 mL mesitylene and added one after the other to a green solution of 0.5 mmol $\{Fe[N(SiMe_3)_2]_2\}_2$ (376.5 mg) in 20 mL mesitylene in a Fischer Porter bottle. The bottle was then pressurized with $H_2$ (2 bar) and placed in an oil bath at 150°C for 48h under vigorous magnetic stirring. After 48h, the reaction was stopped and the NPs were recovered by decantation assisted by a magnet, and washed 3 times (3x10 mL) with toluene and 3 times (3x10 mL) with THF. The NPs were then dried under vacuum.[2]

Carbidization of Fe(0) nanoparticles

In the glovebox, the above synthesized Fe(0) NPs (50 mg, 0.45 mmol of iron) were dispersed in mesitylene (9 mL) in a Fischer Porter bottle, and the mixture was pressurized with CO / $H_2$ (2 bar / 2 bar) at 150°C for 120 h. At the end of the reaction, the NPs were recovered by decantation assisted by a magnet and were washed 3 times with toluene (3x5 mL). The NPs (ca. 75 wt% Fe) were then dried under vacuum.[2]

### *ICNPs@$Cu_2Cr_2O_5$ (12 wt% ICNPs loading)*

In the glovebox, the above synthesized ICNPs (7 mg) and $Cu_2Cr_2O_5$ (53 mg, fine powder with particle sizes of ca. 1 $\mu$m) were dispersed in THF (2 mL) in a Fischer Porter bottle, sealed and sonicated for 5 minutes, then dried under vacuum. The ICNPs@$Cu_2Cr_2O_5$ were recovered in the glovebox in form of a black powder. For stability reasons, the catalyst was finally treated using magnetic induction (350 kHz, 45 mT), thereby 'fixing' the ICNPs to the $Cu_2Cr_2O_5$ surface. Therefore ICNPs@$Cu_2Cr_2O_5$ (60 mg) in a Fischer Porter bottle under argon were placed in the magnetic coil for 1 h, then recovered in the glovebox.

# Catalytic study

### ***Conventional heating***

In a typical experiment, ICNPs@$Cu_2Cr_2O_5$ (30 mg) and the substrate (0.1 mmol) were dispersed in heptane (0.5 mL) in a Fischer Porter bottle inside the glovebox, then sealed and pressurized with $H_2$ (3 bar). The Fischer Porter bottle was immersed in an oil bath of desired temperature for a fixed reaction time. At the end of the reaction, the catalyst was recovered by decantation assisted by a magnet, washed twice with 1 mL heptane and dried under vacuum. Samples of the reaction solution were analyzed with GC-FID using tetradecane as internal standard.

### ***Magnetic induction heating***

#### Batch experiments

In a typical experiment, ICNPs@$Cu_2Cr_2O_5$ (30 mg) and the substrate (0.1 mmol) were dispersed in heptane (0.5 mL) in a Fischer Porter bottle inside the glovebox, then sealed and pressurized with $H_2$ (3 bar). The Fischer Porter bottle was placed in the coil under an alternating magnetic field (350 kHz, electric current adjustable between 0-25 A, with corresponding field strengths of ≈ 0-95 mT), At the end of the reaction, the catalyst was recovered by decantation assisted by a magnet, washed twice with 1 mL heptane and dried under vacuum. To the collected GC samples tetradecane was added as an internal standard and after dilution with acetone the samples were injected into the GC-FID for analysis.

#### Kinetic study

Time profiles:

Similar protocol, but samples were collected over time. At given reaction times, the Fischer Porter bottle was entered in the glovebox and samples of about 20 mg reaction solution were taken with a pipette and weighed into a GC vial for analysis. The Fischer Porter bottle was re-pressurized with $H_2$ (3 bar) and re-placed in the coil for further reaction.

#### Recycling experiments

Similar protocol. At the end of a first reaction, the catalyst was separated from the reaction solution by decantation assisted by a magnet and washed twice with 1 mL heptane. Then, fresh portions of substrate and heptane were added, and the reaction was started for another cycle.

### *Continuous flow miniplant*

The continuous flow miniplant with magnetic induction heating consists of a high pressure HPCL glass column as a tube reactor, PEEK and stainless steel tubing, a HPLC pump, and a series of pressure and temperature transducers, as shown in Figure S7. HPLC pump supplies the organic feed. $H_2$ is dosed into the system via a massflow control. Organic reagents and $H_2$ are mixed in a Tee piece, then the fluid flows through the glass tube reactor where it is subjected to electromagnetic field. For comparable experiments using conventional heating method, the mixed fluid is preheated by trace heating, and then heated by trace heating in the reactor as well. The system pressure is controlled at its outlet using a BPR and the product is collected at this point.

### *Continuous flow experiments*

In a typical experiment, ICNPs@$Cu_2Cr_2O_5$ (500 mg) and glass beads (1 mm diameter, 2 g) were mixed and packed in a glass tube reactor inside the glovebox. This reactor (length of catalyst zone 5.4 cm, total volume 4.24 mL) was then installed in the miniplant and pressurized with $H_2$ (5 bar). Substrate 1 (0.05 mol.$L^{-1}$ in heptane) was pumped (0.1 g.$min^{-1}$, 0.15 mL.$min^{-1}$, liquid residence time = 8.2 min) and mixed into a flowing stream of $H_2$ (0.001 g.$min^{-1}$, 2.47 mL.$min^{-1}$) that enters the catalyst bed in form of bubbles prior to magnetic field in the glass tube reactor. Once the system was stable, the alternating magnetic field started. The global temperature was monitored by an infrared camera. The crude products was directly analyzed by on-line GC over time.

For these experiments, a large excess of $H_2$ was used in order to avoid having a $H_2$ concentration gradient along the reactor bed. As a result, a full conversion of substrate **1** to **1c** corresponds to a $H_2$ conversion of ca. 3%. This is also very similar to the excess of $H_2$ used in the batch reactions.

<u>Determination of the liquid residence time in the catalyst bed:</u>

The reactor was packed with $Cu_2Cr_2O_5$ and glass beads in the same way as for the catalytic experiments described above. The liquid volume in the catalyst bed was estimated experimentally by adding carefully weighed amounts of heptane to the packed reactor until filling and wetting of the catalyst bed. This procedure was repeated three times, giving an average liquid volume of 1.23 mL. This value is very close from what can be calculated assuming a close-packing of equal spheres by the glass beads (26% of the total reactor volume, *i.e.* 1.10 mL).

At a liquid flow rate of 0.15 mL.$min^{-1}$, the liquid residence time is thus 8.2 min.

## Supplementary Tables and Figures

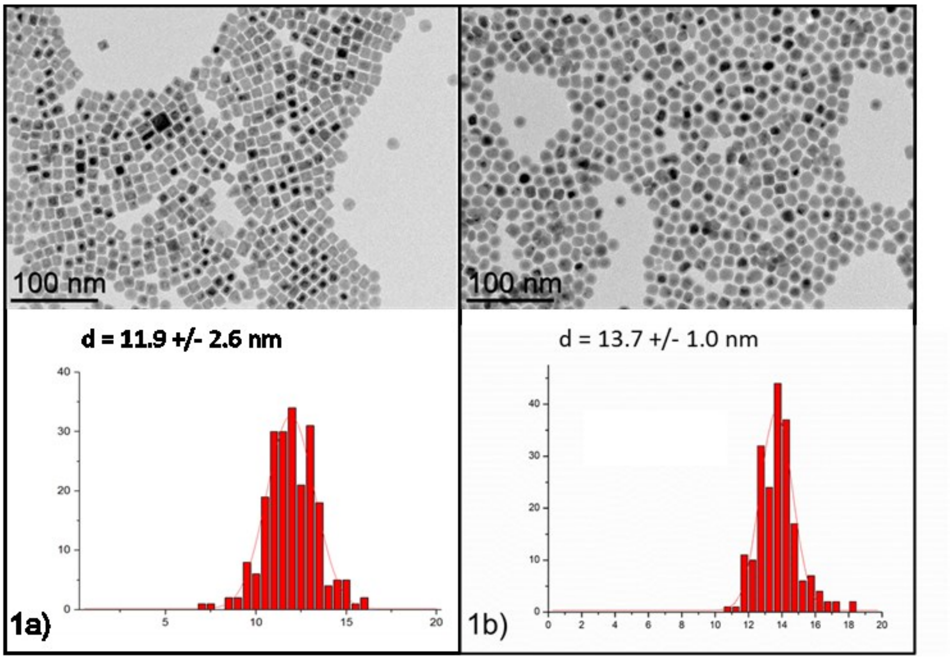


**Figure S1:** TEM images of 1a) Fe(0) NPs and 1b) ICNPs obtained from carbidization of Fe(0) NPs.

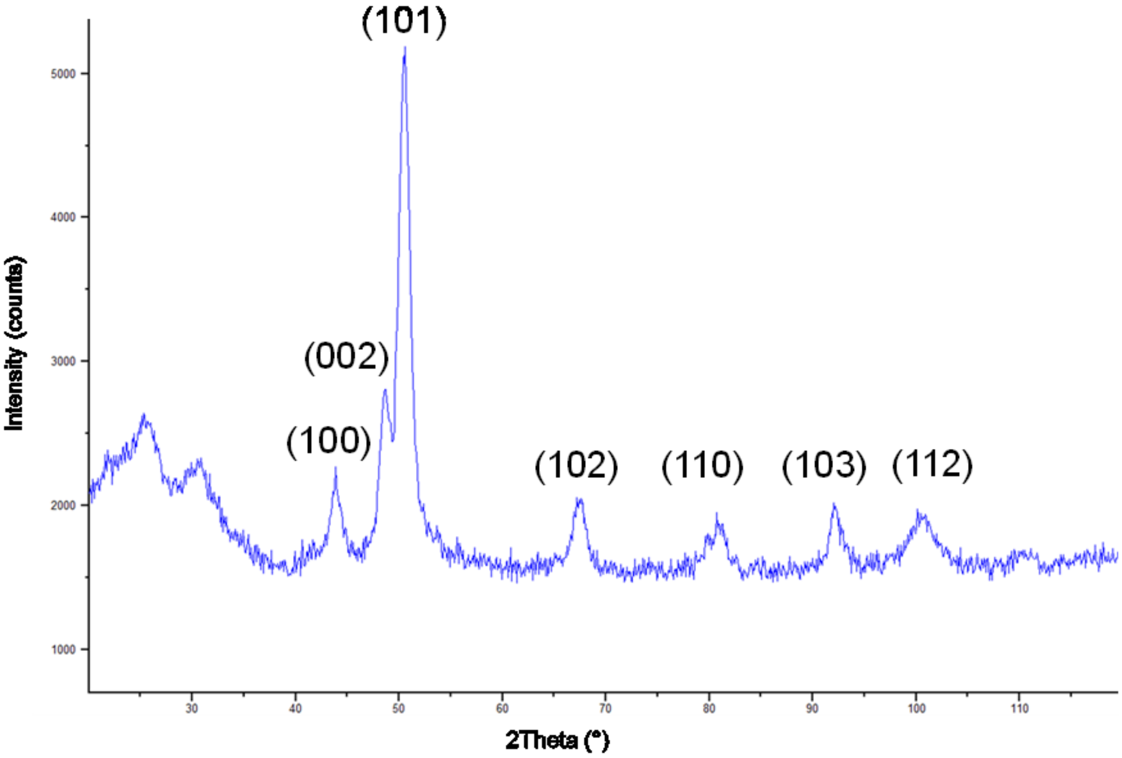


**Figure S2:** XRD pattern of ICNPs (pseudo-hexagonal $Fe_{2.2}C$) obtained from carbidization of Fe(0) NPs.

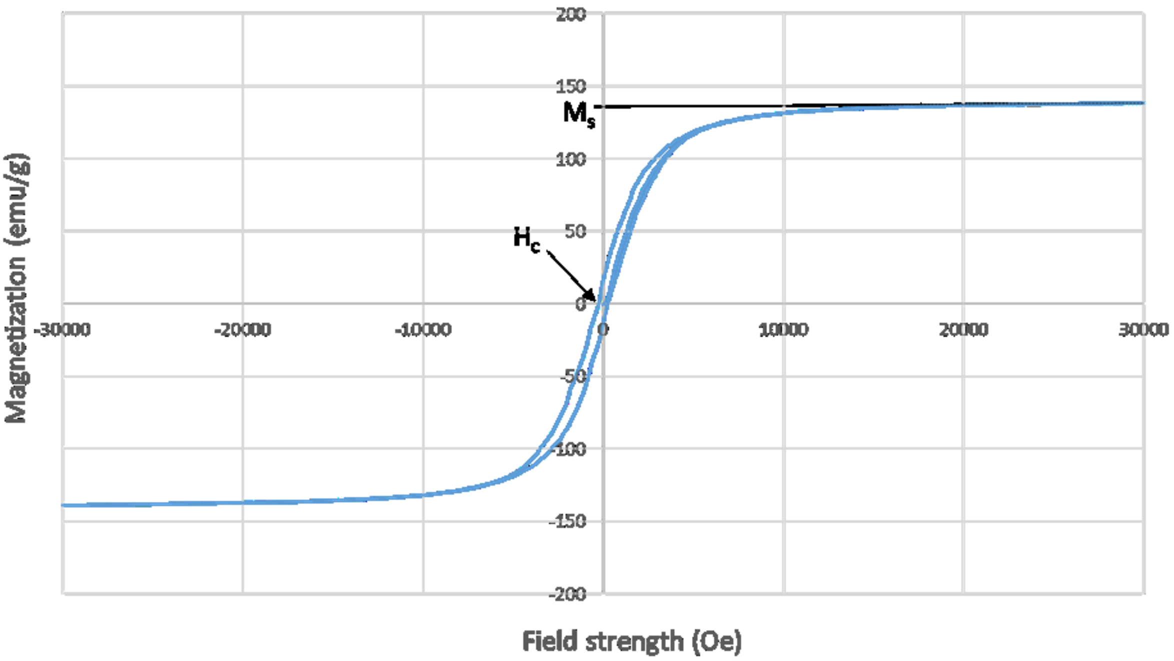


**Figure S3:** Plot of ICNPs magnetization against applied field strength obtained from VSM measurements. $H_c$ = coercivity, $M_s$ = saturation magnetization.

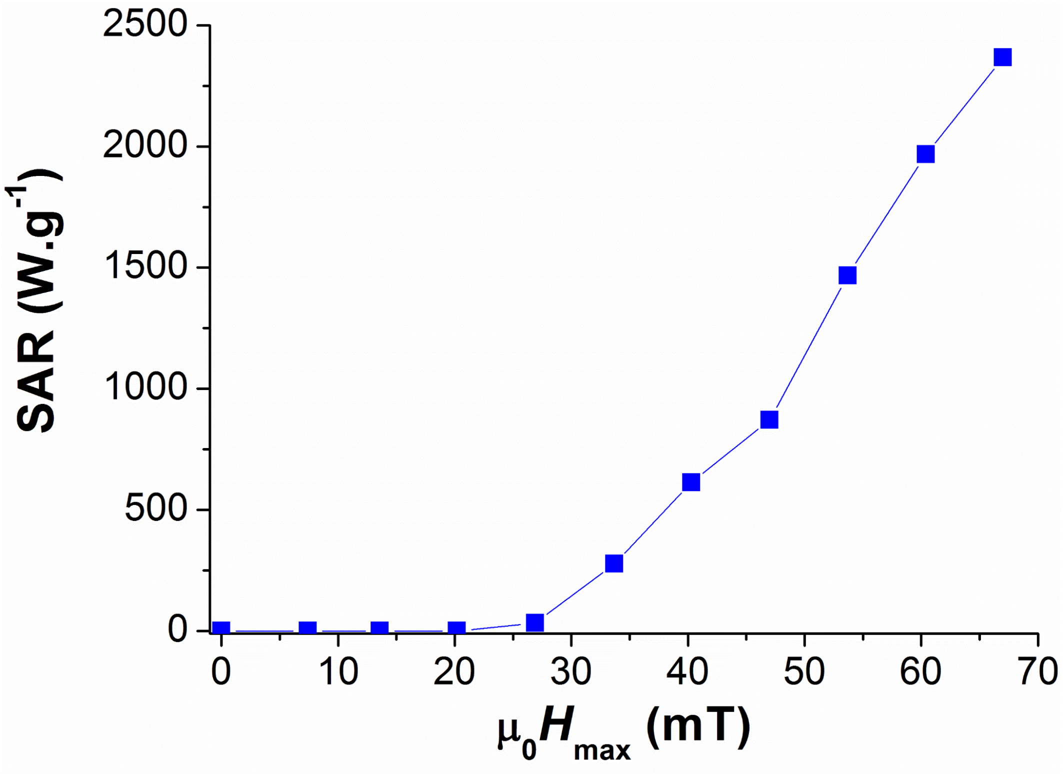


**Figure S4:** Plot of specific absorption rate of ICNPs against applied field strength obtained from calorimetry measurements of ICNPs at 100 kHz. For a detailed measurement protocol, see ref 2.

**Table S1:** Elemental composition of ICNPs@$Cu_2Cr_2O_5$ in wt% determined by ICP-OES.

| ICNPs@$Cu_2Cr_2O_5$ | wt% Fe | wt% Cu | wt% Cr |
|---|---|---|---|
| before catalysis | 8.9 | 36.6 | 29.9 |
| after catalysis (1 cycle) | 8.1 | 36.2 | 29.8 |
| after catalysis (10 cycles) | 8.0 | 36.3 | 30.0 |

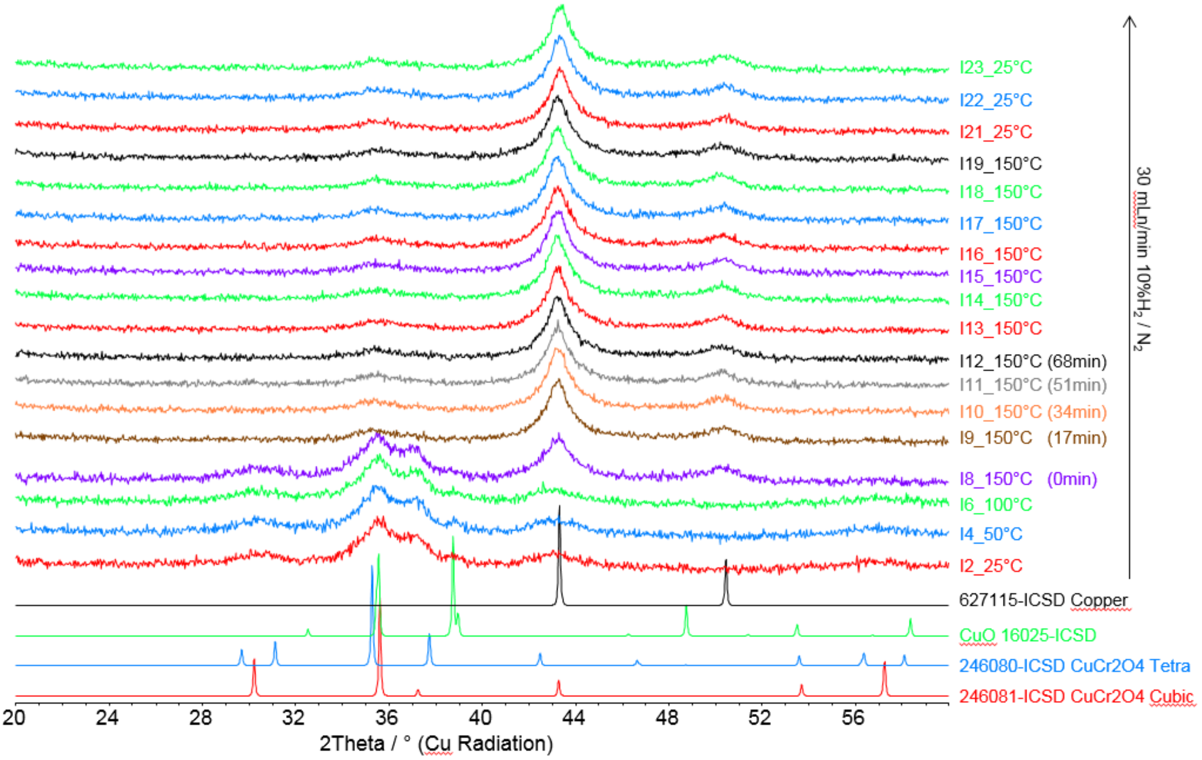


**Figure S5:** XRD spectra of $Cu_2Cr_2O_5$ recorded during in situ reduction at 30 mL/min of 10% $H_2/N_2$ over a temperature range of 25-150 °C. Reference patterns for Cu(0) and $Cu_2Cr_2O_5$ (consisting of mixed cubic and tetra $CuCr_2O_4$ and CuO phases) shown below.

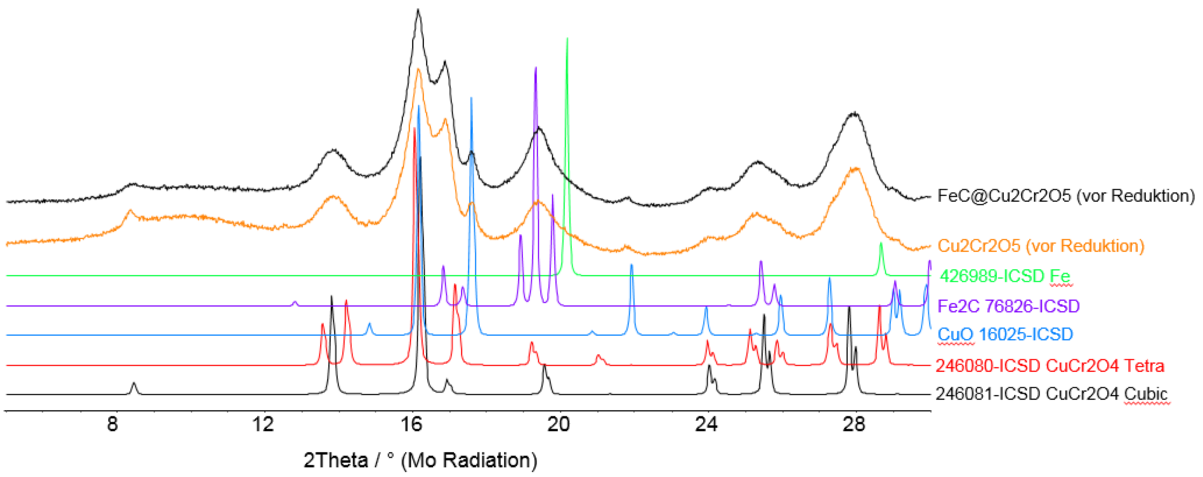


**Figure S6:** XRD spectra of $Cu_2Cr_2O_5$ in comparison to ICNPs@$Cu_2Cr_2O_5$ (FeC@$Cu_2Cr_2O_5$), both recorded before reduction. Reference patterns for $Cu_2Cr_2O_5$ (consisting of mixed cubic and tetra $CuCr_2O_4$ and CuO phases) and for Fe and $Fe_2C$ shown below.

**Table S2:** Correlation of coil power to local temperatures created at solvent/ICNP/$Cu_2Cr_2O_5$ interface

| Coil power (%) | Field amplitude $\mu_0H_{max}$ (mT) | Global solution temperature (°C) | Estimated local catalyst temperature |
|---|---|---|---|
| 10 | 33 | r.t. | - |
| 20 | 42 | 40 | - |
| 30 | 50 | 50 | - |
| 40 | 59 | 65 | T local > 98 °C[a] |
| 50 | 64 | 80 | T local > 110 °C[b] |
| 60 | 71 | 90 | - |
| 80 | 80 | 110 | T local > 165 °C[c] |

All results obtained with 30 mg ICNPs@$Cu_2Cr_2O_5$ (12 wt% ICNPs loading) in FP bottle. Global solution temperature estimated using temperature probe and infrared camera. Local temperature at solvent/ICNP/$Cu_2Cr_2O_5$ interface estimated from local boiling/gas bubble formation of respective solvent. Solvents tested: a) heptane (boiling point = 98 °C), b) toluene (boiling point = 110 °C), c) mesitylene (boiling point = 165 °C).

**Table S3:** Reference experiments performed with pure $Cu_2Cr_2O_5$ and pure ICNPs

| | ICNPs@$Cu_2Cr_2O_5$ (a) | ICNPs (b) | $Cu_2Cr_2O_5$ (c) |
|---|---|---|---|
| Yield of **1c** (*classical heating*) | 97% | 0% | 97% |
| Yield of **1c** (*magnetic heating*) | 96% | 0% | 0% |

Reactions performed in Fischer-Porter-Bottle, with 0.1 mmol furfuralacetone in 0.5 mL heptane under 3 bar $H_2$ without stirring for 3 h. Catalyst quantities used were a) 30 mg ICNPs@$Cu_2Cr_2O_5$ (containing respective amounts of 3.5 mg ICNPs and 26.5 mg $Cu_2Cr_2O_5$), b) 3.5 mg ICNPs, c) 26.5 mg $Cu_2Cr_2O_5$. **1c** = furfuryl alcohol from conversion of furfuralacetone. Classical heating: 120°C. Magnetic heating: 64 mT, 350 kHz.

**Table S4:** Screening of different ICNPs loadings on $Cu_2Cr_2O_5$.

| Entry | ICNPs loading (wt%) | Conversion (%) | Yield **1a** | Yield **1c** |
|---|---|---|---|---|
| 1 | 5 | 13 | 13 | 0 |
| 2 | 12 | >99 | 9 | 91 |
| 3 | 20 | >99 | 4 | 95 |

Reactions performed in Fischer-Porter bottle, with 30 mg ICNPs@$Cu_2Cr_2O_5$ + 0.1 mmol **1** in 0.5 mL heptane under 3 bar $H_2$ for 2 h without stirring. Product yield determined by GC-FID using tetradecane as internal standard.

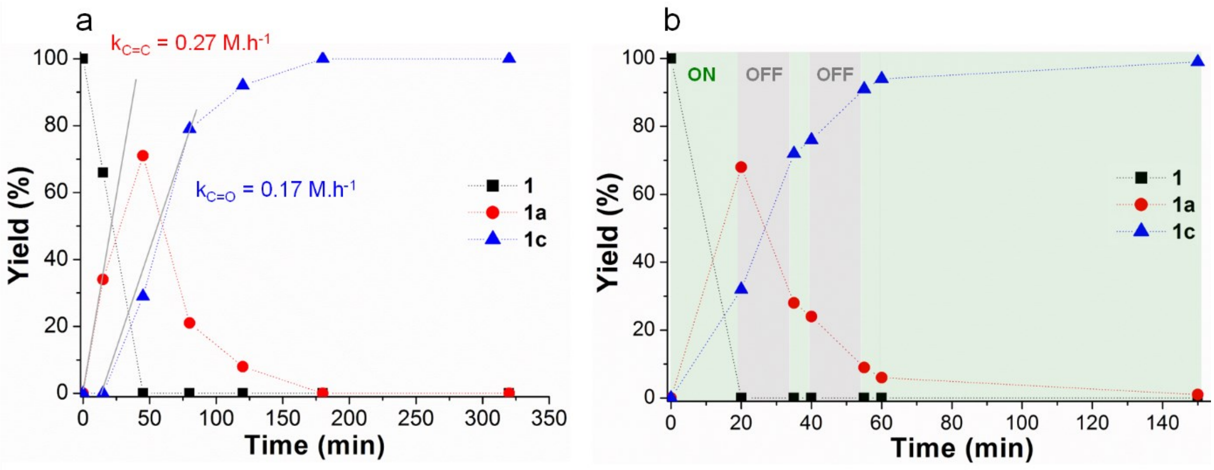


**Figure S7:** : Kinetic study of the hydrogenation of **1** using ICNPs@$Cu_2Cr_2O_5$ and conventional heating at 120°C). a) time profile; b) time profile recorded while regularly switching ON and OFF the power supply of the heating plate (green zones = power ON, grey zones = power OFF). Reactions performed in a Fischer-Porter bottle, with 30 mg ICNPs@$Cu_2Cr_2O_5$ + 0.1 mmol substrate in 0.5 mL heptane under 3 bar $H_2$ without stirring. Yields determined by GC-FID using tetradecane as internal standard.

**Table S5:** Hydrogenation of aromatic ketones using ICNPs@$Cu_2Cr_2O_5$ and conventional heating.

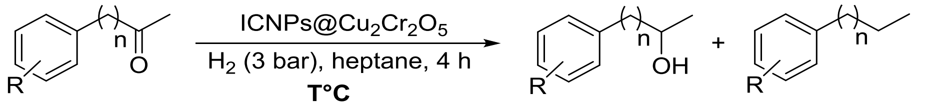


| Entry | Substrate | T°C | Conversion | Product Yield (%)[a] |
|---|---|---|---|---|
| 1 | 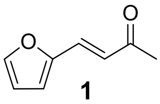 1 | 80 | 0 | 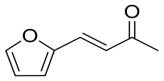 |
| | | 120 | >99 | 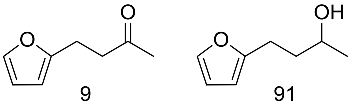 9, 91 |
| 2 | 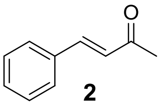 2 | 80 | 0 | 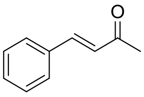 |
| | | 150 | >99 | 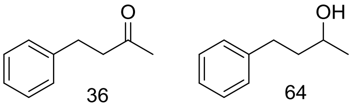 36, 64 |
| 3 | 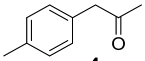 4 | 80 | 0 | 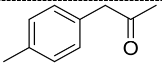 |
| | | 150 | 91 | 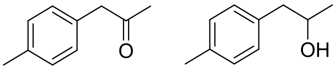 9, 91 |
| 5 | 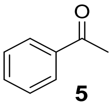 5 | 80 | 0 | 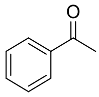 |
| | | 120 | >99 | 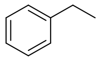 >99 |
| | | 150 | >99 | 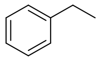 >99 |
| 6 | 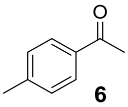 6 | 80 | 0 | 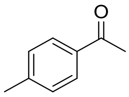 |
| | | 150 | >99 | 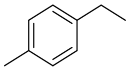 >99 |
| 7 | 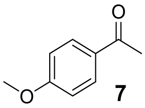 7 | 80 | 0 | 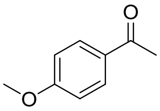 |
| | | 150 | >99 | 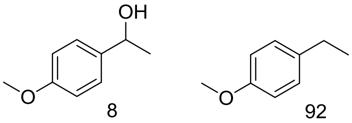 8, 92 |
| 8 | 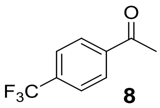 8 | 80 | 0 | 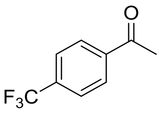 |
| | | 150 | >99 | 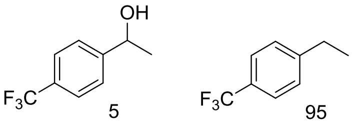 5, 95 |

Reactions performed in Fischer-Porter bottle, with 30 mg ICNPs@$Cu_2Cr_2O_5$ + 0.1 mmol **1** in 0.5 mL heptane under 3 bar $H_2$ for 4 h without stirring. Product yield determined by GC-FID using tetradecane as internal standard.

**Table S6:** Recycling study with ICNPs@$Cu_2Cr_2O_5$ prepared without heat treatment to anchor the ICNPs.

| Entry | Cycle | Conversion (%) | Yield **1a** | Yield **1c** |
|---|---|---|---|---|
| 1 | 1 | >99 | 4 | 96 |
| 2 | 2 | >99 | 27 | 73 |
| 3 | 3 | >99 | 42 | 58 |

Reactions performed in Fischer-Porter bottle, with 30 mg ICNPs@$Cu_2Cr_2O_5$ + 0.1 mmol **1** in 0.5 mL heptane under 3 bar $H_2$ for 2 h at 64 mT without stirring. Product yield determined by GC-FID using tetradecane as internal standard.

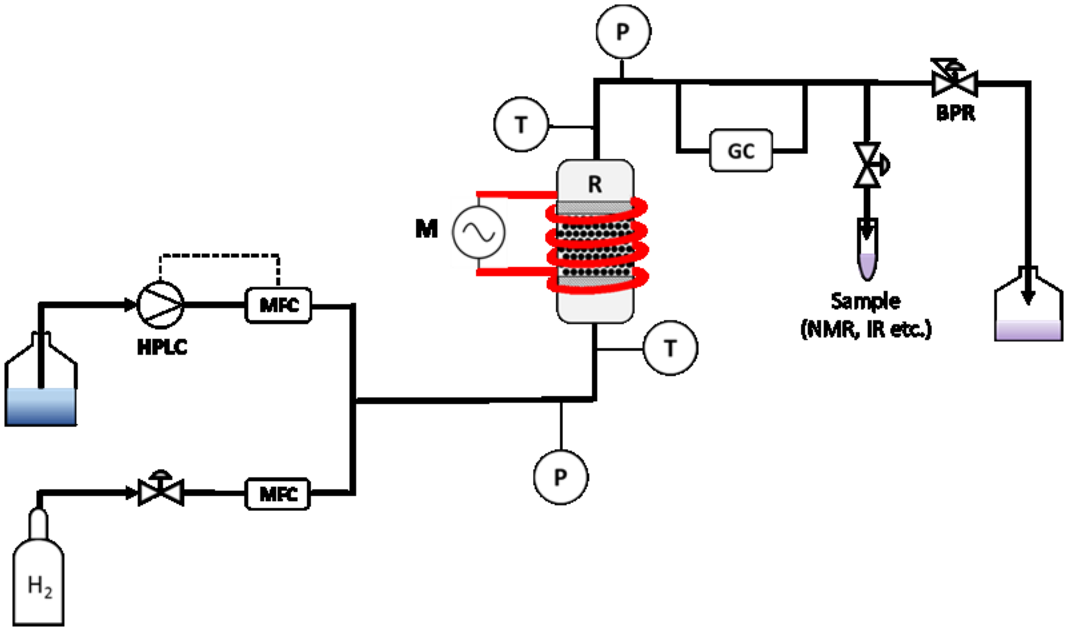


**Figure S8:** Schematic of the continuous flow miniplant used. Substrate 1 is dissolved in heptane which is pumped by HPLC (a Knauer BlueShadow 40P HPLC pump); $H_2$ is delivered by MFC (a massflow control Bronkhorst FG-201CV); R: borosilicate glass tube reactor with PEEK connections (Omnifit HiT columns); M: magnetic coil (Ultraflex Heat induction system UPT-n5). For comparable experiment with conventional heating, a self-designed tracing heating is used instead of M; GC-FID (Shimadzu GC-2030 with a CP WAX 52 CB column) on-line analyze the products; Where a manual sample is required, an extra sample tap is reserved; BPR: back pressure regulator (GCE BP301); P (Wika S-11 pressure transducer) monitors system pressure, T (thermal couple) monitors system temperature.

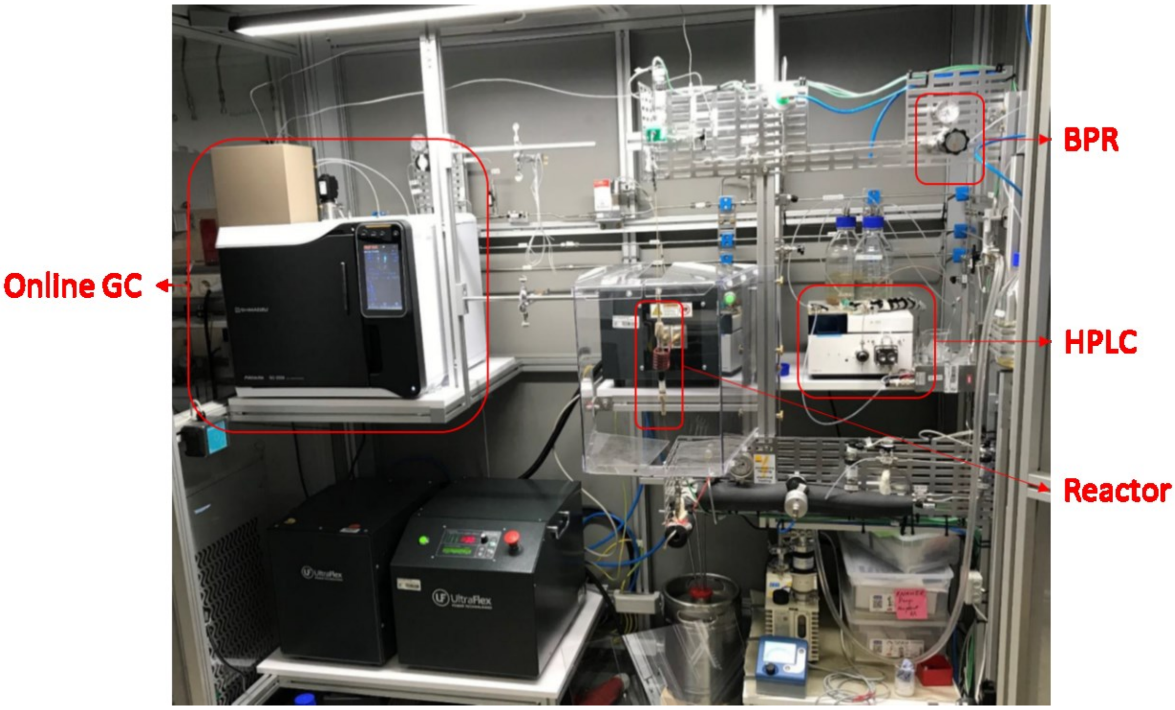


**Figure S9:** Picture of the continuous flow set-up. GC = Gas Chromatograph. BPR = Back Pressure Regulator.

**Table S7:** Continuously operated hydrogenation of furfuralacetone (**1**) using ICNPs@$Cu_2Cr_2O_5$ under various conditions.

| $\mu_0H_{max}$ (mT) | Global T °C | Conversion (%) | Product Yield (%) | | |
|---|---|---|---|---|---|
| | | | 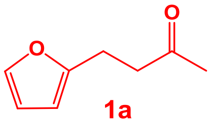 **1a** | 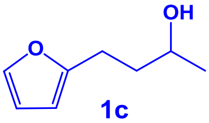 **1c** | 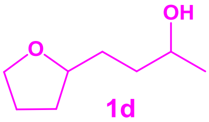 **1d** |
| 33 | 103 | >99 | 15 | 83 | 2 |
| 42 | 112[a] | >99 | 10 | 88 | 2 |
| 50 | 123 | >99 | 21 | 75 | 4 |
| - | 120[b] | 0 | 0 | 0 | 0 |

Reactions performed in continuous miniplant with 500 mg ICNPs@$Cu_2Cr_2O_5$ catalyst. Substrate **1** (0.05 mol.$L^{-1}$ in heptane) was pumped with a flow rate of 0.1 g.$min^{-1}$ and mixed into a flowing stream of $H_2$ (0.001 g.$min^{-1}$). WHSV = 0.1 $h^{-1}$ (0.1 g.$min^{-1}$). System pressure was kept at 5 bar. Product yield determined by a calibrated online GC-FID. [a] Temperature at the outlet of the reaction zone (30 cm above the reactor): 25°C. [b] conventional heating at 120°C.

# Isolated Yields

## Hydrogenation of furfuralacetone (**1**)

ICNPs@$Cu_2Cr_2O_5$ (30 mg) and **1** (0.1 mmol, 13.6 mg) were dispersed in heptane (0.5 mL) in a Fischer Porter bottle inside the glovebox, then sealed and pressurized with $H_2$ (3 bar). The Fischer Porter bottle was placed in the coil under an alternating magnetic field (350 kHz, 64 mT). After 4 h, the catalyst was recovered by decantation assisted by a magnet, washed twice with 1 mL heptane and dried under vacuum. The reaction mixture as well as the washing solution were combined and evaporated under vacuum, giving product **1c** as a pure compound (13.1 mg, 95%).

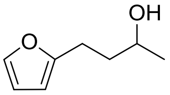

$^1$H NMR (400 MHz, $CDCl_3$) $\delta$ (ppm): 7.30 (1H), 6.28 (1H), 6.01 (1H), 3.86 (1H), 2.75 (m, 2H), 1.82-1.78 (m, 2H), 1.22 (d, 3H).

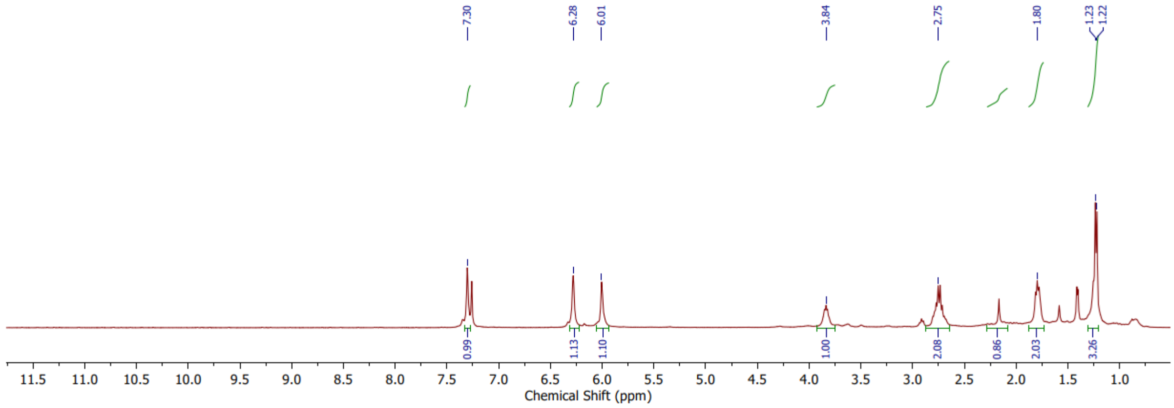


$^{13}$C NMR (100 MHz, $CDCl_3$) $\delta$ (ppm): 155.8, 141.0 110.2, 104.9, 67.4, 37.4, 24.4, 23.6.

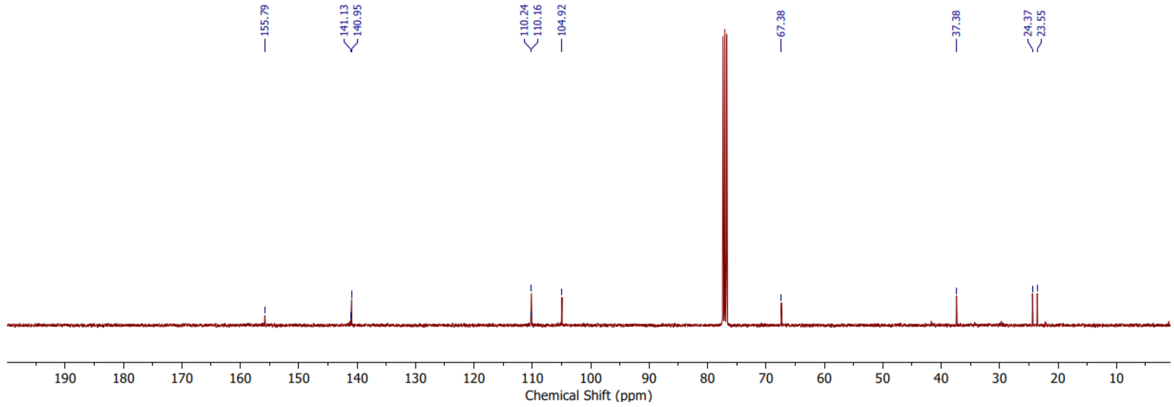

<u>Hydrogenation of benzylideneacetone (**2**)</u>

ICNPs@$Cu_2Cr_2O_5$ (30 mg) and **2** (0.1 mmol, 14.6 mg) were dispersed in heptane (0.5 mL) in a Fischer Porter bottle inside the glovebox, then sealed and pressurized with $H_2$ (3 bar). The Fischer Porter bottle was placed in the coil under an alternating magnetic field (350 kHz, 64 mT). After 8 h, the catalyst was recovered by decantation assisted by a magnet, washed twice with 1 mL heptane and dried under vacuum. The reaction mixture as well as the washing solution were combined and evaporated under vacuum, giving the corresponding aromatic alcohol as a pure compound (14.6 mg, 98%).

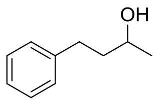


$^1$H NMR (400 MHz, $CDCl_3$) $\delta$ (ppm): 7.35-7.31 (m, 2H), 7.25-7.23 (m, 3H), 3.88 (m, 1H), 2.81-2.72 (m, 2H), 1.85-1.78 (m, 2H), 1.45 (d, 1H), 1.31 (s, 1H), 1.27 (d, 3H).

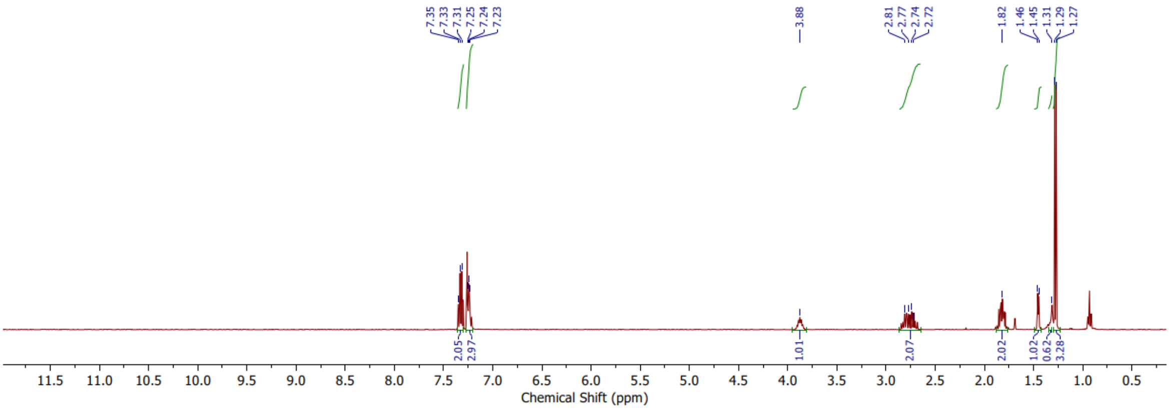


$^{13}$C NMR (100 MHz, $CDCl_3$) $\delta$ (ppm): 142.1, 128.4, 125.9, 67.6, 40.9, 32.2, 23.7.

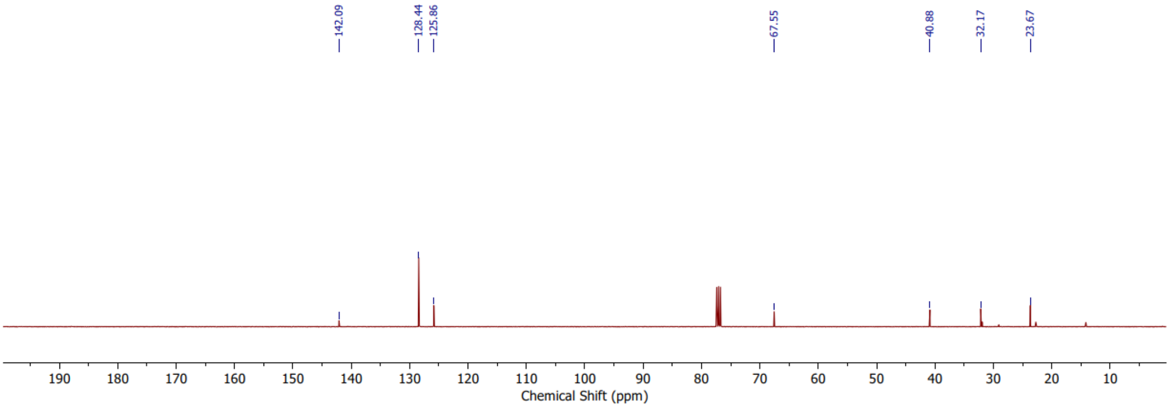

## References


1. Strohmann, M., Bordet, A., Vorholt, A. J. & Leitner, W. Tailor-made biofuel 2-butyltetrahydrofuran from the continuous flow hydrogenation and deoxygenation of furfuralacetone. *Green Chem.* **21,** 6299–6306 (2019).

2. Bordet, A. *et al.* Magnetically Induced Continuous $CO_2$ Hydrogenation Using Composite Iron Carbide Nanoparticles of Exceptionally High Heating Power. *Angew. Chem. Int. Ed.* **55,** 15894–15898 (2016).

3. Deutsch, K. L. & Shanks, B. H. Active species of copper chromite catalyst in C-O hydrogenolysis of 5-methylfurfuryl alcohol. *J. Catal.* **285,** 235–241 (2012).

4. Rao, R., Dandekar, A., Baker, R. T. K. & Vannice, M. A. Properties of Copper Chromite Catalysts in Hydrogenation Reactions. *J. Catal.* **171,** 406–419 (1997).
